# MOB: Pivotal Conserved Proteins in Cytokinesis, Cell Architecture and Tissue Homeostasis

**DOI:** 10.3390/biology9120413

**Published:** 2020-11-24

**Authors:** Inês L. S. Delgado, Bruno Carmona, Sofia Nolasco, Dulce Santos, Alexandre Leitão, Helena Soares

**Affiliations:** 1 CIISA-Centro de Investigação Interdisciplinar em Sanidade Animal, Faculdade de Medicina Veterinária, Universidade de Lisboa, 1300-477 Lisboa, Portugal or ines.delgado@ulusofona.pt (I.L.S.D.); or sbnarciso@estesl.ipl.pt (S.N.); dulcesantos@fmv.ulisboa.pt (D.S.); alexandre@fmv.ulisboa.pt (A.L.); 2 Faculdade de Medicina Veterinária, Universidade Lusófona de Humanidades e Tecnologias, 1749-024 Lisboa, Portugal; 3 Escola Superior de Tecnologia da Saúde de Lisboa, Instituto Politécnico de Lisboa, 1990-096 Lisboa, Portugal; bruno.carmona@estesl.ipl.pt or; 4 Centro de Química Estrutural–Faculdade de Ciências da Universidade de Lisboa, 1749-016 Lisboa, Portugal

**Keywords:** MOB, cell polarity, cytokinesis, tissue homeostasis, proliferation, morphogenesis, hippo, MEN

## Abstract

**Simple Summary:**

Multicellular organisms develop from a single cell into a multitude of cell types organized in tissues and organs. Organisms grow at a coordinated pace until reaching mature size. From this point, tissues and organs retain a constant size despite cell renovation. This depends on cell-cell communication and the constant integration of multiple signals, being regulated by several signaling pathways. The Hippo pathway stands out as it is also present in unicellular organisms. Some Hippo components control key cellular processes in unicellular organisms, like cell division and the perpetuation of cell morphology. Intrigued by this, we set out to understand the interplay between unicellular and multicellular Hippo regulation from the perspective of the most ancient proteins from this pathway, hereafter designated Monopolar spindle One Binder protein (MOB). We reviewed MOB functions along the tree of life to uncover its ancestral role, aiming to understand the origin of multicellularity. MOB-regulated cellular processes like division and death are essential for organism growth and tissues maintenance. The research on MOB of unicellular organisms highlighted the role of these proteins in the regulation of cell number and shape, critical issues for the maintenance of healthy tissues in multicellular organisms. This knowledge allows a better understanding of some human diseases like cancer.

**Abstract:**

The MOB family proteins are constituted by highly conserved eukaryote kinase signal adaptors that are often essential both for cell and organism survival. Historically, MOB family proteins have been described as kinase activators participating in Hippo and Mitotic Exit Network/ Septation Initiation Network (MEN/SIN) signaling pathways that have central roles in regulating cytokinesis, cell polarity, cell proliferation and cell fate to control organ growth and regeneration. In metazoans, MOB proteins act as central signal adaptors of the core kinase module MST1/2, LATS1/2, and NDR1/2 kinases that phosphorylate the YAP/TAZ transcriptional co-activators, effectors of the Hippo signaling pathway. More recently, MOBs have been shown to also have non-kinase partners and to be involved in cilia biology, indicating that its activity and regulation is more diverse than expected. In this review, we explore the possible ancestral role of MEN/SIN pathways on the built-in nature of a more complex and functionally expanded Hippo pathway, by focusing on the most conserved components of these pathways, the MOB proteins. We discuss the current knowledge of MOBs-regulated signaling, with emphasis on its evolutionary history and role in morphogenesis, cytokinesis, and cell polarity from unicellular to multicellular organisms.

## 1. Introduction

A MOB protein was first identified in 1998 by Luca & Winey [1], following a two-hybrid screen in budding yeast *Saccharomyces cerevisiae* that detected a monopolar spindle one (Mps1) binder protein. It was not until 2005 that Lai et al. (2005) [2] identified the first metazoan *Mob* gene, designated *Mob* as tumor suppressor (*Mats*), in *Drosophila melanogaster*. Since then, research efforts have identified *Mob* genes in further species expanding this family in the eukaryotic lineage. *Mob* genes are found in variable numbers with fungi and fly, typically possessing three to four genes, while humans have up to seven [3,4,5].

**Figure 1:**
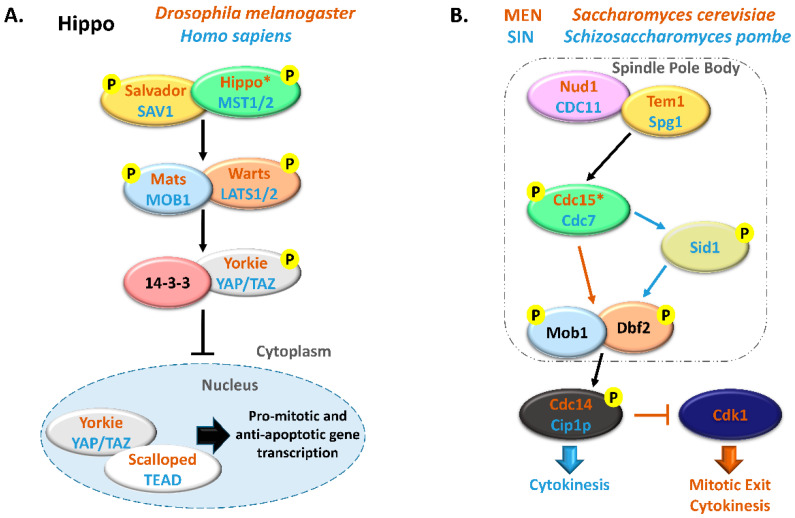
Comparison between Hippo and MEN/SIN pathways. (**A**) Schematic representation of the Hippo pathway in *Drosophila melanogaster* (orange) and in *Homo sapiens* (blue). (**B**) Schematic representation of the Mitotic Exit Network (MEN) in *Saccharomyces cerevisiae* (orange) and Septation Initiation Network (SIN) in *Schizosaccharomyces pombe* (blue). In these pathways, ortholog proteins are represented with oval nodes of the same color (yellow, Salvador/SAV1/Tem1/Spg1; green, Hippo/MST1/2/Cdc15/Cdc7; blue, Mats/MOB1/Mob1; orange, Warts/LATS1/2/Dbf2). * There are some controversies in the literature regarding whether to consider Cdc15 and Hippo as orthologous. This scheme is in accordance with Hergovich, 2017 [17].

The MOB family proteins are constituted by highly conserved eukaryote kinase adaptors that are often essential both for cell and organism survival. MOB proteins were first characterized as regulators of ploidy maintenance, helping to ensure proper chromosome segregation before mitotic exit and allowing the transition from mitosis to cytokinesis [1]. This family is also necessary for maintaining cell polarity and morphology during cell division [6,7]. In multicellular organisms, MOBs have been mostly characterized as tumor suppressors and agents of morphogenesis, frequently by regulating GCKII STE20 and NDR kinases in the Hippo signaling pathway. Notably, the Hippo pathway interplays with various signaling pathways, like Wnt, mTOR, Notch, Hedghog, and the STRIPAK complex [8,9,10]. These pathways control a myriad of cellular processes and their activity is largely dependent on intercellular communication. In metazoans, the canonical Hippo signaling pathway comprises a core kinase cascade that includes Salvador/SAV1 and Mats/MOB1 as signal adaptors. When the Hippo pathway is activated, Hippo/MST1/2 kinase together with Salvador/SAV1 activate by phosphorylation Mats/MOB1 in a complex with the Warts/LATS1/2 kinase that in turn phosphorylates Yorkie/YAP/TAZ transcriptional co-activators, preventing its translocation into the nucleus and avoiding the transcriptional activation of target genes (Figure 1). Since YAP/TAZ induce the transcription of anti-apoptotic and proliferation-associated genes, such as BIRC5/surviving, BIRC2/cIAP1, and MCL1, Hippo signaling activation results in tumor suppression [11,12]. Alternative interactions between adaptor proteins, GCKII STE20, and NDR kinases result in non-canonical Hippo pathway signaling. MOB adaptor proteins, GCKII STE20 kinases, and NDR kinases are highly conserved protein families from yeast to metazoans. In multicellular eukaryotes, MOB proteins have been mostly characterized in the context of canonical Hippo signaling. However, these proteins also regulate cell biology processes in these species, namely mitotic exit, centrosome duplication and chromosome segregation [13,14,15]. Significantly, most core Hippo pathway components present homologous proteins in unicellular eukaryotes. In these organisms, MOB co-activators, GCKII STE20 kinases, and NDR kinases have been mainly characterized in the context of the MEN/SIN, both ensuring the correct genetic material distribution and cytokinesis (Figure 1). Interestingly, both MEN and SIN networks have a Cdc14 phosphatase as an effector protein, a highly conserved protein also present in multicellular eukaryotes [16].

Extensive reviews on MEN and Hippo signaling pathways have been published recently and we would like to direct the readers to them [5,18,19,20,21]. MEN/SIN and Hippo pathways have been mostly considered independent signal transduction cascades but the existence of several highly conserved proteins in these pathways suggests a shared functionality. With this review, we intend to explore the hypothesis that the Hippo pathway represents an evolution and functional expansion of the MEN/SIN. We will focus on the most conserved proteins of these pathways, the MOB kinase regulators. We will summarize MOB protein functions and regulatory processes in an evolutionary perspective from unicellular to multicellular eukaryote organisms including a phylogenetic and amino acid residue analysis of key eukaryote MOB sequences.

## 2. MOB Proteins in Multicellular Eukaryotes

The MOB family proteins are currently classified into four isotypes that tend to be functionally different within each species: MOB1, MOB2, MOB3, and MOB4/Phocein. Some species, particularly more complex organisms, also present sub-isotypes that tend to be functionally similar or redundant within each species, e.g., MOB1A and MOB1B. In several publications MOB proteins have been referred as MOB3/Phocein [9,22,23,24,25], however this nomenclature has been reviewed based on *Homo sapiens* designations (described by Gundogdu et al., 2019) [18] and during this revision we will refer to these proteins using the updated designation of MOB4/Phocein. This chapter focuses on functional evidence of MOB protein activity and regulation in multicellular eukaryotes, including multicellular fungi. A later chapter will be focused on MOB proteins in unicellular eukaryotes.

### 2.1. Functions of MOB Proteins

#### 2.1.1. Tissue Homeostasis: MOB as Regulators of Cell Proliferation and Apoptosis

**Figure 2:**
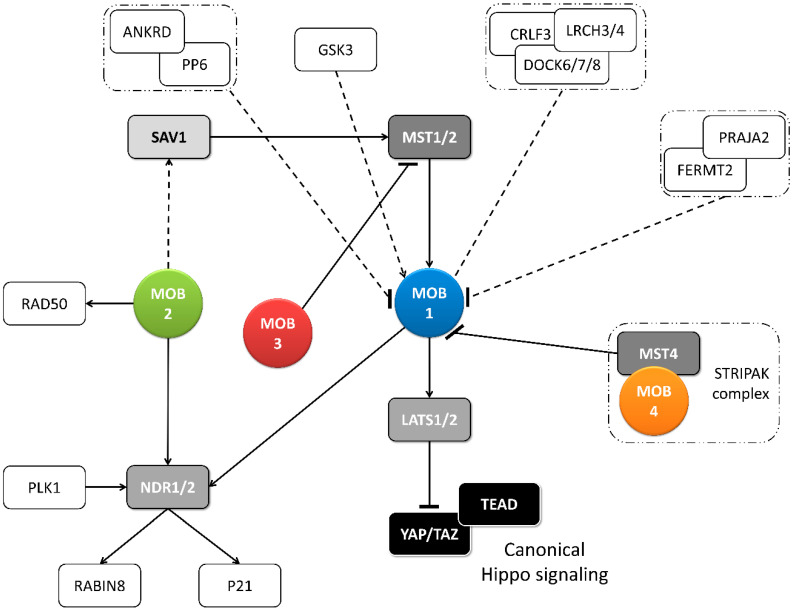
Metazoan MOB proteins present extensive regulation between isotypes. The canonical Hippo pathway is activated by upstream signals resulting in MST1/2 phosphorylation (which may be mediated by SAV1) and MOB1-LATS1/2 activation, causing YAP/TAZ phosphorylation and cytoplasm retention. The lack of YAP/TAZ transcriptional signaling results in a tumor suppressing effect. However, MOB proteins present several activities beyond canonical Hippo signaling. These include non-canonical Hippo signaling through different interactions with GCKII STE20 or NDR kinases, direct MOB stimulation by upstream signals and direct stimulation by MOBs of non-Hippo proteins. This intricate network results in direct and indirect MOB to MOB regulation. PLK1 regulates mitotic spindle orientation through NDR1 phosphorylation which results in NDR1 binding shifting from MOB1 to MOB2, favoring canonical Hippo activation [48]. NDR1/2 also regulate P21 and RABIN8 [49,50]. MOB3 is a MOB1 antagonist by inhibiting MST1 [36]. MOB4/Phocein-MST4, part of the STRIPAK complex, also antagonizes MOB1, by disrupting MOB1-MST1/1 binding [34]. MOB1 interacts in a HsMOB1-PPP6R1/2/3-ANKRD28 complex which appears to inhibit MOB1 mediated Hippo activation and in a DOCK6/7/8-CRLF3-LRCH3/4 complex, in a phosphorylation dependent manner [51,52]. Both PP6 phosphatase and DOCK6-8 promote actin cytoskeleton polarization signaling via RAC1. The FERMT2-PRAJA2 complex inhibits Hippo signaling by promoting MOB1 ubiquitin-proteasome degradation [40]. MOB1 is stimulated by GSK3β, a signaling hub involved in Wnt, mTOR, and Notch signaling [53]. MOB2 interacts with RAD50 stimulating the DNA damage response [54]. MOB2-SAV1 interaction was detected in *G. gallus* [42]. Notably, PP6, DOCK6, FERMT2 and GSK3 are proteins involved in cell-cell junctions. Colored boxes represent Hippo pathway members while other proteins are represented by non-colored boxes. Protein complexes are identified by an interrupted box with dashes and dots lines. Arrows represent activation while dashes represent inhibition. Lack of arrow or dash indicates an uncertain effect. Full lines represent well-established interactions. Interrupted lines represent less documented interactions.

MOB proteins are tumor suppressors playing a critical role in regulating tissue homeostasis maintenance. In *D. melanogaster*, it was shown that DmMOB1 (Mats) controls cell proliferation and apoptosis through interaction with DmLATS (Warts), and its lethal depletion phenotype is rescued by HsMOB1 showing function conservation from invertebrates to vertebrates [2,26]. Indeed, *H. sapiens*, HsMOB1 also presents tumor suppressor activity, by phosphorylating HsLATS1, which can be triggered by HsMST1/2 phosphorylation but this is not essential (Figure 2 and Table 1) [27,28]. HsMOB1 tumor suppressor activity involves apoptotic signaling through Hippo pathway activation [29]. DmMOB1 tumor suppressor activity independent of DmMST (Hippo) phosphorylation also occurs in *D. melanogaster* [26]. Both HsMOB1 and HsMOB2 have been implicated in tissue growth suppression in cancer development [30,31,32,33]. Conversely, Chen et al. (2018) [34] showed that the cancer promoter complex HsMST4-HsMOB4/Phocein negatively regulates the tumor-suppressing complex HsMST1-HsMOB1 in pancreatic cancer. HsMOB4/Phocein and HsMST4 integrate the Striatin-interacting phosphatase and kinase (STRIPAK) complex [35]. The STRIPAK complex also includes the protein phosphatase PP2A and regulates vesicular trafficking, microtubule cytoskeleton and morphogenesis [9]. HsMOB3 also shows tumorigenic properties in glioblastoma cells by suppressing HsMST1 activity [36]. In *Mus musculus*, MmMOB1 functions as a tumor suppressor and tissue homeostasis factor as a member of Hippo signaling, namely by controlling apoptotic signaling in keratinocytes [10,37,38,39]. MmMOB1 also participates in renal homeostasis, MmMOB1 mediated Hippo activation, through MmLATS1 and MmYAP phosphorylation, is associated with diminished renal fibrosis [40]. The Wnt (wingless integrated) pathway is also activated but seems to have an opposite association. In *Canis familiaris*, CfMOB1-CfLATS1 Hippo signaling appears to regulate photoreceptor homeostasis [41]. In *Gallus gallus*, GgMOB2 interacts with GgSAV1 which acts as a growth suppressor through Hippo signaling [42]. *Arabidopsis thaliana* AtMOB1 regulates plant growth and development, and tissue homeostasis through interaction with AtMST, known as SIK1 [43,44,45,46]. In other angiosperms, *Medicago sativa*, MsMOB1 also regulates cell proliferation [47]. However, Ms*Mob1* does not complement budding yeast MOB1 temperature sensitive growth phenotype. In the fungus *Neurospora crassa*, Nc*Mob1* gene deletion results in overall reduced mycelium growth [3]. Nc*Mob2* gene deletion exhibited phenotypes similar to Nc*Mob1* but with less intensity. Nc*Mob4* gene deletion resulted in a very mild decrease in tissue growth. *Aspergillus nidulans* AnMOB4/Phocein, which integrates the STRIPAK complex, also showed tumor suppressor properties [22]. Overall, several MOB isotypes are involved in the regulation of cell proliferation and apoptosis and its activation induces inhibition or promotion of tissue growth.

#### 2.1.2. Morphogenesis: A MOB Function in Various Species and Cell Types

MOB proteins are also important cell polarity and tissue morphogenesis regulators (Table 1). *D. melanogaster* DmMOB2 interacts with DmNDR (Trc) and is necessary for wing hair, photoreceptor and neuromuscular junction morphogenesis [66,67,68]. DmMOB4/Phocein is also necessary for synapse morphogenesis and microtubule organization [69]. In *H. sapiens*, HsMOB2 loss of function mutations are associated with defective cortical development [59]. Interestingly, a similar phenotype is produced by mutations in cell adhesion protocadherins FAT4-DCHS1 which is compensated by Yap knockdown [73]. *M. musculus* MmMOB1 controls lung morphogenesis through YAP/TAZ regulation and neuritogenesis independent of YAP [53,62]. MmMOB1 mediated neuritogenesis is stimulated by GSK3β (glycogen synthase kinase3β) [53]. Notably, GSK3 functions as a signaling hub, integrated into Wnt, mTOR (mammalian target of rapamycin, and Notch pathways [74]. MmMOB2 is necessary for neuritogenesis and cortical development, including ciliogenesis [59,64]. MmMOB2 RNAi and overexpression studies showed this protein’s role in neuritogenesis is synergistic with MmNDR2 and affects the actin cytoskeleton [64]. Contrary to *H. sapiens*, *M. musculus* MmDCHS1 mutation phenotype could not be compensated by YAP knockdown [59]. MmMOB4/Phocein regulates dendritic arborization in neuronal development through STRIPAK complex signaling [25]. In *G. gallus*, GgSAV1-GgMOB2 interaction also regulates follicle development [42]. *A. thaliana*, AtMOB1 is necessary for plant structural development of stem and root [43,44,46]. Also, in fungi, MOB proteins participate in morphogenesis. In *N. crassa* NcMOB1 is necessary for septum and aerial mycelium formation and conidiation with Nc*Mob1* gene deletion resulting in increased hyphae branching despite decreased mycelium growth. NcMOB2 controls hyphae polar tip extension through regulation of the NDR kinase NcCOT1 [3]. NcMOB4/Phocein is essential for vegetative cell fusion and consequent fruiting body formation in a way unrelated to NDR signaling. NcMOB1 and NcMOB4/Phocein both participate in fruiting body morphogenesis but NcMOB1 activity occurs by interaction with NcLATS (DBF2) while NcMOB4/Phocein integrates the STRIPAK complex [3,24]. Moreover, NcMOB4/Phocein and SmMOB4/Phocein of the Ascomycete *Sordaria macrospora* also control vegetative cell fusion [3,23]. These data show that the involvement of MOB proteins in morphogenesis is not exclusive of a specific MOB isotype, but that different MOB isotypes participate in this process (Table 1).

#### 2.1.3. Cell Cycle Progression: MOBs as Regulators of Mitosis, Cytokinesis, and Centrosome Biology

**Figure 3:**
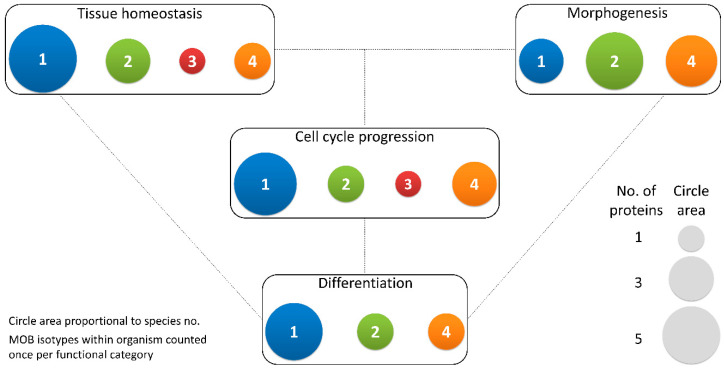
Different MOB isotypes present similar functions. MOB functions were divided into 4 categories: tissue homeostasis, morphogenesis, differentiation and cell cycle progression (includes mitosis, meiosis, cytokinesis, and centrosome biology). Circle areas are proportional to the number of proteins counted in the literature related to these functions, as is illustrated in the protein count examples at the bottom right. MOB protein isotypes for each organism were counted only once per function category (variants within protein isotypes were not considered). It is obvious that there is no one MOB isotype responsible for a particular function. Various MOB isotypes present similar functions, be it in distinct species or in distinct tissues in the same species. In fungi, NcMOB1, ChMOB2, and SmMOB4/Phocein, all participate in conidiation, ascosporogenesis, and meiosis [3,4,23]. In *M. musculus,* MmMOB1, MmMOB2, and MmMOB4/Phocein are necessary for neuronal development while MmMOB1 also promotes lung morphogenesis [25,53,62,64]. The data also evidence a higher abundance of studies on MOB1 proteins, which are the more represented in each category, except morphogenesis. Colors representing MOB isotypes: 1—blue, 2—green, 3—red, 4—orange.

Several core components of the Hippo pathway have been implicated in the regulation of eukaryotic cell cycle progression, including MOB proteins (Figure 2 and Figure 3) [17]. DmMOB1 null mutants present aberrant chromosome segregation during embryogenesis [15] while DmMOB4/Phocein depleted cells fail to properly assemble the spindle pole [70]. DmMOB4/Phocein also integrates the STRIPAK complex and is necessary for PP2A regulated axonal transport of autophagosomes [71]. DmMOB1 localizes to the cytoplasm and nucleus, but also to the centrosome [15]. HsMOB1 is required for mitotic exit and its depletion results in prolonged telophase [13]. The same is true for its binding partner HsLATS1/2. HsMOB1 is necessary for correct positioning of the mitotic regulator chromosomal passenger complex to the spindle midzone during anaphase [56]. Correct mitotic spindle orientation is regulated through NDR1 phosphorylation by PLK1 which results in NDR1 binding shifting from HsMOB1 to HsMOB2 [48]. HsMOB1/HsMOB2 competitive binding to NDR1 was also observed in centrosome duplication [14]. HsMOB2 binding to NDR seems to inhibit its activation which occurs through phosphorylation [60]. HsMOB1 has also been implicated in centrosome disjunction by interfering with NEK2 centrosome localization [57]. HsMOB1 also regulates correct cell abscission and cytokinesis [55]. HsMOB1 knockdown cells show increased motility immediately after telophase/cytokinesis and persist connected by long intercellular bridges [55]. Also, MOB1 depletion results in centriole separation which supports the idea that MOB1 is required for centriole rejoining at the end of mitosis. Interestingly, components of the Hippo pathway, HsSAV1 and MST2 cooperate with the NEK2 kinase to regulate centrosome disjunction [57]. Additionally, HsMOB1 interacts with serine/threonine phosphatases PP6 and an ankyrin repeat–containing protein ANKRD, forming a HsMOB1-PPP6R1/2/3-ANKRD28 complex. Also, interactions with leucine-rich repeats and calponin homology domain–containing proteins LRCH, cytokine receptor–like factor 3 and dedicator of cytokinesis proteins DOCK forming a HsDOCK6/7/8-CRLF3-LRCH3/4 complex were described [51,52]. Time point analysis suggests the PP6 complex may inhibit HsMOB1 mediated Hippo activation. HsMOB1 is mostly localized in the cytoplasm and to a lesser extent in the cytoplasmic membrane, but it also localizes to various cell cycle associated structures, namely centrosome, kinetochores in early mitosis and spindle midzone in late mitosis [13,14,56,61]. HsMOB2 induces G1/S cell cycle arrest in response to DNA damage, independently of NDR kinases [54]. Coincidently, HsMOB2 localizes to the nucleus [14,61]. HsMOB3, a cytoplasmic protein, prevents high cell density growth inhibition by downregulating HsMST1 mediated apoptotic signaling in glioblastoma cells [14,36]. Similarly to human, in mouse MmMOB1 regulates centrosome duplication in keratinocytes, through the regulation of MmLATS and MmYAP [37]. MmMOB1 also interacts with MmDOCK8 in thymocytes, stimulating MmRAC1 actin cytoskeleton polarization signaling [63]. MmMOB1-MmMST1/2 mediate MmRhoA GTP charging and MmRAC1 signaling stimulation. *C. familiaris* CfMOB1 is also necessary for correct mitosis in photoreceptor cells [41]. In plants *A. thaliana* and *M. sativa*, AtMOB1 and MsMOB1 participate in apoptotic signaling during meiotic microsporogenesis and macrosporogenesis and also regulate cytokinesis [43,46,47]. Supporting its role in cytokinesis MsMOB1 is cytoplasmic, but localizes to the cell plate during septum formation and at spindle microtubule structures related to cytokinesis [47]. AtMOB1 is present at the cytoplasm, cytoplasmic membrane, and nucleus [43,44]. *A. thaliana* Hippo/MST1/2 homolog SIK1 complements *S. cerevisiae* Ste20 deletion in mitotic exit and SIK1 was shown to bind to AtMOB1 [45]. In conclusion, MOB proteins play critical roles in accurate cell division and cytokinesis and, besides other cellular localizations, tend to localize at the centrosome when this structure is present, a localization that is shared by other Hippo components (Table 1).

#### 2.1.4. Differentiation and Stem Cell Maintenance: MOB in a Crossroad between Morphogenesis, Tissue Homeostasis, and Cell Cycle Regulation

In *Drosophila* DmMOB1 is essential for embryonic development [15]. This becomes evident after both zygotic and maternal DmMOB1 depletion that results in non-viable embryos indicating a probable role for DmMOB1 in stem cell differentiation. In mouse, MmMOB1 is necessary for embryonic stem cell differentiation into the three germ layers, an activity that is dependent on YAP regulation [65]. MmMOB1 is also involved in bronchioalveolar cell differentiation and alveolar stem cell maintenance through YAP/TAZ regulation [62]. Surprisingly, this last study did not find evidence that MmMST or MmLATS kinases were also involved in YAP/TAZ regulation. The plant AtMOB1 is necessary for sexual development, namely during sporogenesis and gametogenesis [43,44]. AtMOB1 depleted cells present reduced meristem length and cell number, suggesting a role not only in cell differentiation but also in stem cell maintenance, as documented in mouse [43,44,46]. AtMOB1 depleted cells also present a substantially reduced seed output [43]. The protein localizes to meiocytes, supporting its participation in meiosis. Interestingly, in *M. sativa* MsMOB1 localizes to the meristem in a cell cycle dependent manner [47]. Fungi MOB proteins are also involved in the differentiation of cells for sexual development and the meiotic process. The NcMOB1, *Colletotrichum higginsianum* ChMOB2 and SmMOB4/Phocein are involved in conidiation, ascosporogenesis, and meiosis [3,4,23]. NcMOB2 exhibited similar properties as NcMOB1 in conidiation, but not in ascosporogenesis and meiosis [3]. ChMOB2 interacts with ChNDR, known as Cbk1 [4]. *A. nidulans* AnMOB4/Phocein is also necessary for ascospore production through meiosis [22].

### 2.2. Regulation of MOB Proteins

#### 2.2.1. Post-Translation Modifications of MOBs

MOB1 can be regulated by different phosphorylation events coordinated by diverse kinases. In 2008, it was described for the first time that the MST1/2 kinases catalyzed the phosphorylation of HsMOB1 Thr12 and Thr35 amino acid residues and that these post-translation modifications (PTMs) slowed down the cell cycle progression [27]. Later, a knowledgebase dedicated to mammalian PTMs reported the existence of additional phosphorylation modifications in HsMOB1 amino acid residues, namely in Tyr26, Ser23, and Ser38 [75]. Recently, it was described that focal adhesion kinase (FAK) regulates YAP by phosphorylating on HsMOB1 Tyr26 residue. This modification results in the dissociation of the functional HsMOB1/LATS complex, thus preventing Hippo-dependent inhibition of YAP [76]. It is still unclear whether this regulation could also affect the HsMOB1/NDR complex [18]. On the other hand, the HsMOB1 Ser23 and Ser38 phosphorylation function remains unresolved. However, the HsMOB1 Ser23 represents a possible target site for ATM serine/threonine kinase [77], suggesting a possible link between HsMOB1 and the DNA damage response (DDR). In fact, HsMOB1 and HsMOB2 were detected in RNAi screens to identify modulators of genome integrity [78,79]. HsMOB2 was also identified as a suppressor of homologous recombination in a genome-wide RNAi-based screen and was shown to interact with RAD50, resulting in recruitment of a RAD50 DNA damage sensor complex to damaged chromatin [54,80]. Until now, there is no information concerning HsMOB2 phosphorylation. However, HsMOB2 contains several putative ATM phosphorylation sites, which may be related to the DDR function [77].

Additionally, MmMOB1 Ser146 phosphorylation by the GSK3 kinase (glycogen synthase kinase) was described in the context of neurite outgrowth downstream of the PTEN-GSK3β axis, controlling neurite outgrowth after spinal cord injury [53].

Similar to HsMOB1, HsMOB3A, HsMOB3B, and HsMOB3C are modified by several phosphorylation events in distinct amino acid residues: Thr15, Thr26, and Ser38 from HsMOB3A; Thr25 and Thr77 from HsMOB3B; Thr14, Thr25, and Ser37 from HsMOB3C [75]. Although the role of these modifications is unclear, the sequence motifs surrounding Thr15 and Ser38 of HsMOB3A are quite similar to Thr12 and Thr35 of HsMOB1 [18], raising the hypothesis that HsMOB3A is phosphorylated on these residues by MST1/2 similarly to what has been described for HsMOB1 [27]. The Ser37 residue of HsMOB3C may represent an ATM targeting site [77].

The phosphorylation of HsMOB4/Phocein, without any associated regulatory role, has also been observed at Ser147 and Tyr141 residues [75]. Comparable to the phosphorylation of Ser23 of HsMOB1, of Ser37 of HsMOB3C and putative phosphorylation sites of HsMOB2, the Ser147 phosphorylation of HsMOB4/Phocein is probably performed by the DDR-linked ATM kinase [77], and will be required for the DNA damage signaling.

No other post-translation modifications were identified in MOB proteins, except that HsMOB2 is ubiquitylated, with ubiquitin linked through at Lys23, Lys32, and Lys131 residues [81,82]. The function of these unique PTMs in HsMOB2 are unknown since the fate of a ubiquitylated protein is largely determined by the type of ubiquitin modification with which it is decorated (for review Deol, 2019 [83]). In human cells, the focal adhesion molecule FERMT2 drives Hippo signaling inhibition by interacting with HsMOB1 and the E3 ligase praja2 and promoting HsMOB1 ubiquitin-proteasome degradation [53].

#### 2.2.2. Transcriptional and Post-Transcriptional Regulation of MOBs

Data on transcriptional regulation of *Mob* genes is scarce, but alteration of DNA methylation patterns has been proposed as a possible mechanism to affect regulation of the levels of MOB proteins and is often associated to cancer. Studies of DNA methylation patterns revealed that lysine demethylase 2B (KDM2B) directly bound to the promoter region of Hs*Mob1* gene, inhibited and promoted pancreatic ductal adenocarcinoma progression [84]. Furthermore, in a study concerning methylation in human breast cancer in response to resveratrol, HsMOB1 promotor presented methylation changes in triple-negative breast cancer cells [85]. HsMOB3B transcripts were not downregulated in public data sets, it was also observed that the HsMOB3B promoter region is hypermethylated in a significant number of prostate cancer samples [86].

In the last years, non-coding RNAs (ncRNAs) have emerged as critical molecules for the post-transcriptional regulation of gene expression. For example, miRNAs have been considered key regulators of target mRNAs by driving its degradation or translational repression. In the case of the Hippo pathway, multiple miRNAs have been described to target members of this via in cancer [87,88]. In this context, only three miRNAs were described to target Hs*Mob1* genes: (i) miR-664a-3p, promotes cell proliferation and invasion in gastric cancer [89]; (ii) miR-135b, promotes migration and invasiveness in lung cancer [90]; and (iii) miR-181c, promotes tumor progression pancreatic cancer [91]. Beyond cancer, exosomal miRNAs are important for intercellular communications and functional regulation in recipient cells, being important for several other diseases. Exosomes from human umbilical cord mesenchymal stem cells (HUCMSCs) are effective in inhibiting bone marrow mesenchymal stem cell (BMSC) apoptosis and preventing rat disuse osteoporosis (DOP) by the miR1263/RnMOB1/Hippo signaling pathway [92].

These few examples on the transcriptional and post-transcriptional regulation of MOB1/Hippo signaling strengthen the need to further understand the molecular mechanisms that regulate/deregulate this pathway. Certainly, it will bring new insights into how this pathway is regulated allowing to define new diagnostic strategies and open new avenues for therapies in, e.g., cancer and developmental diseases.

## 3. MOB Proteins in Unicellular Organisms: The Roots of Multicellularity?

MOB proteins have been studied in metazoans for more than one decade now, which consolidated their role as signal adaptors that can interact with different kinase families playing critical roles in the Hippo signaling pathway and Hippo-like signaling pathway [5]. Most studies were carried out in multicellular organisms, strongly suggesting that the Hippo pathway, in crosstalk with other signaling pathways, presents a conserved role throughout the bilaterian animals, thus being a probable hallmark of multicellularity. To trace the evolutionary roots of the Hippo signaling pathway, researchers focused their attention on the presence of genomic sequences coding for the main effector of the pathway, the Yorkie (Yki)/YAP. Genome wide analysis and comparative genome approaches associated with heterologous gene expression studies clearly showed that orthologs of several components of the Hippo pathway, including Yki/YAP, were present in cnidarians (e.g., sea-anemone *Nematostella vectensis*), placozoans (e.g., *Trichoplax adhaerens*), and sponges, that are non-bilaterian animals [93,94]. Similar studies identified Yki/YAP homologs in two holozoan protists that are assumed to be the closest unicellular relatives of metazoans, namely the filastereans *Capsaspora owczarzaki* and the choanoflagellate *Monosiga brevicollis* [95]. Noteworthy, the functional analysis of *C. owczarzaki* Hippo components showed that they were able to regulate tissue growth in *Drosophila* by activating Yki/YAP [95]. Based on these data, the authors proposed that the Hippo pathway originated in unicellular organisms within the holozoa, before the divergence of filastereans, choanoflagellates, and metazoans [95]. Later, Ikmi et al. (2014) [96], also using heterologous gene expression assays, showed that Yki/YAP proteins from *M. brevicollis*, *A. queenslandica*, *T. adhaerens*, *N. vectensis,* and their non-phosphorylatable forms (YAP activity is regulated through phosphorylation by Warts/Lats1,2 kinases) were able to regulate growth in *Drosophila*, and to be regulated by phosphorylation, similarly to what is observed for *Drosophila* Yki and human YAP.

Currently, most of the core components of the Hippo pathway (e.g., Mats/MOB1, Hippo/MST1/2, and Warts/LATS1/2) have been identified in the genome of animal lineages that diverged before the bilaterians and seem to have similar functions. This suggest that Mats, Hippo, and Warts are evolutionarily more conserved than their upstream regulators (i.e., Fat/Dachsous/Crumbs) and downstream partners in the via (Yki/YAP/TAZ and Scalopped (Sd)/TEAD) [94]. However, there are still some discrepancies between different studies, for example Yki/YAP was identified in *C. owczarzaki*, but bona fide Yki/YAP orthologue is apparently absent from ctenophore genomes [97]. Nevertheless, atypical sequences may be present in the genome of these organisms. Only TAZ proteins, that are YAP paralogs, were not found in non-vertebrates and seem to have appeared late in evolution. Recently, it was suggested that YAP and TAZ seem to share the same evolutionary origin since TAZ was originated from Yki/YAP during the whole genome duplication in fish, which may explain their redundant roles in the mammalian Hippo pathway [98]. Although Yki/YAP exhibit a high degree of coevolution with Mats/MOB1 [93], this last protein is conserved throughout the eukaryotic lineage, whereas Salvador/SAV1, another adaptor of the core pathway, is only present in choanoflagellates and metazoa [95]. By analyzing the evolutionary history of the Hippo pathway in a variety of unicellular organisms like the ciliate *Tetrahymena thermophila*, the centric diatom *Thalassiosira pseudonana*, the algae *Chlamydomonas reinhardtii*, the flagellated protozoan parasite *Trichomonas vaginalis*, and the excavate *Naegleria gruberi*, Chen et al. (2020) [98] concluded that Mats/MOB1 was the earliest member of the Hippo pathway to be identified in these unicellular species. Accordingly, to this study, the Hippo/MDT1/2 kinase was the next member of this via to emerge and was identified in the Amoebozoa (e.g., *Acanthamoeba castellanii*), Dictyostelia (e.g., *Dictyostelium discoideum*), and Apusozoa (e.g., *Thecamonas trahens*). The Warts/LATS1/2 kinase was firstly identified in the cellular slime mold *Fonticula alba* and in *S. cerevisiae*. The Hippo/Warts/Mats core kinase complex was revealed in the chytrid fungus *Spizellomyces punctatus*, whereas the Yki/Sd or YAP/TEAD transcriptional complex was found in the unicellular choanozoan *Corallochytrium limacisporum*, an organism belonging to the supergroup Opisthokonta and assumed to be important to understand the evolutionary origin of animals and fungi. Finally, a complete Hippo pathway was attained in *C. owczarzaki* [98] which agrees with the data of Sebé-Pedrós et al. (2012) [95]. The ancestor components of the Hippo pathway, (i.e., Mats/MOB1, Hippo/MST1/2, and Warts/LATS1/2) from *T. thermophila*, *A. castellanii*, and *F. alba* not only share key domains and regulatory amino acid residues with *C. owczarzaki*, *Drosophila*, and human orthologs but also present conserved functions in human cells as revealed by heterologous gene expression assays [98].

The presence of a complete Hippo pathway in a unicellular organism is now undoubtedly accepted but its function is still uncertain. In metazoans, Hippo signaling plays critical roles during development and is in crosstalk with other signaling cascades, for example Notch, Hedgehog, and Wnt signaling, all absent in unicellular organisms. Sebé-Pedrós et al. (2012) [95] proposed that the Hippo pathway in unicellular organisms may be involved in the coordination of cell proliferation in response to cell density or cell polarity. This assumption was based on the fact that the metazoan apical proteins Merlin/NF2 (recognized as a critical mediator of contact inhibition of proliferation and regulator of Hippo pathway), Kibra (functions as a scaffold protein in various cell processes, such as cell polarity, cell migration, and membrane trafficking [99]), aPKC kinase (plays a critical role in the regulation of apical-basal polarity in epithelial cells and asymmetrically dividing cells [100]) and Lethal-2-giant larvae (Lgl, makes part of the Scribble cell polarity module involved in cell polarity, control of tissue growth, differentiation and directed cell migration [101]) are encoded in the *C. owczarzaki* genome [95]. Moreover, recent studies in ciliates showed that some Hippo proteins link accurate cell division and cytokinesis to morphology [6]. In unicellular organisms, the maintenance of morphology likely relies more on self-organization than in extrinsic/intrinsic cues as it occurs in metazoa, with the Hippo proteins having a crucial role in the perpetuation of cell pattern and organization. Thus, Hippo signaling research in unicellular organisms may reveal the ancestral regulatory routes of cell division and cell number control in metazoans

Probably, the establishment of a tight regulation control may have contributed to the establishment of multicellularity. Studies in unicellular organisms will allow for the definition of core and pivotal conserved modules of these signaling pathways. Consequently, in the next sections, we will focus our attention on the role of MOB proteins in two groups of unicellular organisms where the functional role of this protein has been largely studied, namely the yeast and members of the alveolate clade.

### 3.1. MOB Proteins in Yeast: From Cell Division to Signaling Pathways

The budding yeast, *S. cerevisiae* divides asymmetrically originating two daughter cells that differ in cell size and specific inheritance of molecules [102]. This asymmetrical division requires cell polarization, and many polarity factors are recruited to the site where cell division will take place, i.e., the bud neck. Interestingly, the fission yeast *Schizosaccharomyces pombe* that present a rod-shape cell grows by polarized tip extension and divides symmetrically by medial fission [103]. Both yeasts do not contain centrioles or basal bodies, but they possess a structure designated by the spindle pole body. This structure is a microtubule-organizing center that can organize the nuclear microtubules required for nuclear positioning, and the cytoplasmic microtubules that are involved in chromosome segregation during cell division [104]. Spindle pole bodies are proteinaceous, multi-layered structures that duplicate only once per cell cycle. In budding yeast *S. cerevisiae*, this structure is permanently embedded in the nuclear envelope whereas in fission yeast it is inserted into the nuclear envelope before mitosis [104].

In *S. cerevisiae*, the essential MOB protein (Mob1p) is an interactor of the Mps1 kinase required for the spindle pole body duplication and control of the mitotic checkpoint [1]. Conditional alleles of Mob1p showed that the protein was necessary for successful mitosis end and maintenance of ploidy. Also, genetic and biochemical studies showed interactions between MOB1 and three genes involved in mitosis completion, namely the GTP exchange factor LTE1 that encodes a daughter specific protein, and the kinases Cdc5, Cdc15, and the Dbf2 and Dbf20 (counterparts of mammalian NDR/LATS kinases) [1,105]. In yeast, these proteins are involved in the regulation of cell cycle transition from mitosis to G1 that implies the degradation of the mitotic cyclins and the inactivation of cyclin-dependent kinase. The major signaling via that regulates these events is the MEN which plays important role in the coordination of the spindle orientation with mitotic exit. The final outcome of MEN is the onset of cytokinesis through the inactivation of the mitotic cyclin Cdk1 [21,106]. Indeed, the MEN is a critical component of the checkpoint that controls the correct spindle position in anaphase [107]. The establishment of MOB1 as an interactor of the MEN kinases and the phenotype of conditional Mob1p mutants of late mitotic arrest, positioned this protein as a component of the MEN [108]. In fact, at late mitosis, Mob1p localizes to spindle pole bodies and the bud neck where Dbf2 kinase is also detected which is consistent with their role in cytokinesis [109]. Ma and coworkers (2001) [110] showed that Cdc15 kinase promotes the exit from mitosis by directly activating the Dbf2 kinase in a Mob1p dependent manner. In turn, the activation of the Cdc15 kinase is dependent on the initial activation of the GTPase Tem1 that occurs at the spindle poles starting the MEN signaling [21,111,112]. Interestingly, the MEN proteins that are required for Dbf2 kinase activity are also necessary for actin ring formation at the bud neck [112]. Finally, the phosphorylation of the Cdc14 phosphatase by the complex Dbf2-Mob1p complex promotes its transport from the nucleolus to the cytoplasm thereby causing a dephosphorylation wave through which cyclin B/cdc34p is inactivated triggering the mitosis exit [113].

In the fission yeast *S. pombe* the SIN signaling cascade is the equivalent to MEN and is essential to regulate the time and place of cytokinesis in connection with cell cycle progression, but is not part of the checkpoint that monitors spindle position [107,114]. SIN via triggers the actomyosin ring constriction and septum formation after chromosome segregation [20,106]. Like in *S. cerevisiae*, the *S. pombe mob1* gene is essential and the protein is located on both spindle pole bodies throughout mitosis. Later in mitosis Mob1p localizes at the medial ring and when the ring starts to constrict it borders the septum [115]. This localization and the phenotypes of the spores lacking Mob1p, which are not viable elongated multinucleated cells, are typical of a role on septation [115]. Also, the Sid2p kinase (the counterpart of *S. cerevisiae* Dbf2p and Dbf20p kinases) localizes at the spindle pole body at all stages of the cell cycle and transiently at the cell division site where medial ring constricts and septation occurs [116]. Sid2p kinase activation and localization are dependent on Mob1p being the Sid2p-Mob1p kinase complex, a key element of this via determining the time and local of cell division (septation) [117,118].

The SIN signaling cascade (Figure 1) is similar to MEN in *S. cerevisiae* and the Hippo signaling pathway in mammalian cells [17]. Indeed, all these signaling pathways possess a conserved core of NDR-family kinases composed of Dbf2p/Mob1p in *S. cerevisiae*, LATS1/2-MOB1/MOB1B in mammals, and Sid2/Mob1p in *S. pombe* (for review Simanis, 2015 [114]). However, the SIN pathway contains an additional kinase module composed of a PAK/GC family protein kinase, Sid1 and its activator Cdc14p, that act downstream of STE-20 family kinase Cdc7p (the fission yeast counterpart of Cdc15p) that interacts with Spg1p (Tem1p in *S. cerevisiae*) and upstream of Sid2p-Mob1p kinase complex regulating the onset of cytokinesis [119]. This module may be involved in the integration of signals from other pathways with those of Cdc7p and Spg1 GTPase to activate the Sid2p-Mob1p kinase complex [114].

Noticeably, most MEN and SIN components localize at the spindle pole body [120]. In SIN signaling during mitosis, some of the components of the via asymmetrically associate with the duplicated spindle pole bodies being SIN hyperactivated on the “new” spindle pole body [121,122,123]. This polarized localization seems to be important for SIN regulation and to shut off the signal cascade after cytokinesis completion [124,125]. Contrary to SIN, MEN is active on the ‘‘old’’ spindle pole body [126], still, its activity is also asymmetric in both spindle bodies. The MEN signaling asymmetry seems to be established through microtubule-bud cortex interactions [127].

Interestingly, the SIN signaling pathway is not exclusive of the unicellular *S. pombe* but most of its components, e.g., Mob1p and the Sid1p and Sid2p kinases are encoded by genes present in the genomes of syncytial ascomycete fungi *N. crassa* and *A. nidulans* [128,129]. However, in these fungi that possess multinuclear hyphal, SIN components localization and how this via is regulated significantly differs from that of yeast [130]. This clearly shows that these conserved signaling networks have evolved to cope with cell morphology diversity.

A second member of the Mobp family is present in both yeast genomes, the nonessential *mob2* gene. In *S. cerevisiae* Mob1p presents a 43% sequence similarity at the amino acid level and 33% identity with Mob2p [1]. Mob2p regulates polarized cell growth through its interaction with the other members of the NDR/LATS kinase family, namely Cbk1p in budding yeast [131] and Orb6 kinase in fission yeast [118]. In fact, in *S. cerevisiae* both Cbk1p and Mob2p localize, at late mitosis, to the bud neck, exactly when the transcription factor Ace2p starts to accumulate in the daughter nucleus. In the nucleus, this factor activates daughter-specific genes and genes involved in mother/daughter separation after cytokinesis [131]. After mitotic exit Mob2p–Cbk1p are required to avoid the daughter nuclear export of Ace2p. Consequently, they participate in the coordination of Ace2p-dependent transcription with MEN activation, establishing a connection between cell morphology and cell cycle transitions with cell fate determination and development [132]. Mob2p in complex with Cbk1p are indeed core components of the regulation of Ace2p and morphogenesis (RAM) network that besides controlling polarized growth also regulates cell separation, mating, maintenance of cell wall integrity, and stress signaling [132,133,134,135,136]. This signaling pathway is conserved among eukaryotes from yeasts to humans, including the pathogenic fungal species (*e.g.*, *Candida albicans*, *Candida glabrata*, *Cryptococcus neoformans*, *Aspergillus fumigatus*, and *Pneumocystis* spp.), where it also plays an important role in the pathogenesis of these organisms [133].

### 3.2. MOB Proteins in the Alveolate: From Cytokinesis to Morphology

Alveolata is a morphologically and ecologically varied clade of organisms that include the Ciliates, Dinoflagellates, and Apicomplexa. These organisms are characterized by the presence of cortical alveoli (membrane-bound sacs underlying the cell membrane) that gives the name to the clade [137]. The alveolate have extraordinarily large and complex cells that can reach millimeters in length.

#### 3.2.1. MOB in Ciliates

Ciliates, that are characterized by the presence of cilia and the separation of the somatic lineage (vegetative macronucleus) from the germ lineage (micronucleus only active during sexual reproduction), demonstrate a tremendous morphological diversity [138]. For example, the ciliate *Tetrahymena* is a complex permanently polarized unicellular organism. This ciliate displays an elaborated cortex characterized by the specific pattern organization of thousands of basal bodies, most harboring motile cilia, and high diversity of microtubule structures [138]. The basal bodies are organized in longitudinal rows that extend from the anterior to the posterior region of the cell. This organization is broken in specific regions by the occurrence of complex structures like the oral apparatus and cytoprocts that create specific cell territories. Therefore, *Tetrahymena* cells present a permanent anteroposterior axis and left-right asymmetry and this organism is an excellent biological model to study development and morphogenetic mechanisms. The complex ciliate cortical organization of *Tetrahymena* cells is perpetuated during the symmetrical division of the ciliate cell. During this event, the continuity of longitudinal rows of basal bodies is interrupted, and a fission furrow develops at the equatorial region of the cell. The cell grows throughout the antero-posterior axis by the addition of new basal bodies to the preexisting longitudinal rows [138,139,140].

Interestingly, in the ciliate *Tetrahymena* vegetative cells, Mob1 accumulates in the basal bodies of the posterior pole creating a gradient through the antero-posterior axis of the cell. When cells divide Mob1 is recruited to the basal bodies that are localized at the cell midzone, just above the region where the cleavage furrow will be established. Thus, Mob1 localizes at the new posterior pole of the anterior daughter cell. The localization of Mob1 at basal bodies is mediated by Sas4 that promotes scaffolds for Mob1 localization to the cell cortex [141]. The *Tetrahymena* Sas4 is required, not only for basal body assembly and maintenance but integrates these events with the surrounding environment linking the perpetuation of cortical organization to cell division [141]. The depletion of Mob1 in *Tetrahymena* causes the abnormal establishment of the cell division plane and cytokinesis arrest. In these cells, as far as the division continues, Mob1 progressively accumulates with CdaI protein at the region where the furrow will be established and the future posterior pole of the anterior daughter cell develops [7,142]. Notably, the CdaI protein is absent in non-dividing cells and starts to localize at the ciliary rows of the anterior half of the cell when division initiates. Also, *Tetrahymena CdaI* mutants [143] phenocopies the depletion of Mob1 [142]. Moreover, the posterior boundary of the CdaI stained region is affected by the ELO1 protein [32] suggesting that the size of this region is regulated by these two proteins [32,142]. In fact, loss-of-function of *Elo1* gene moves the division plane to the posterior pole [32], whereas the depletion of CdaI displaces it to the anterior pole of the cell [32]. Resembling the Mob1 localization, the ELO1 protein localizes at the posterior basal bodies in non-dividing cells and is recruited to the midline when division starts [7,32]. The *Tetrahymena* CdaI and ELO1 proteins are orthologues of the human MST1/2 kinases and the NDR/Lats kinases, respectively, showing that *Tetrahymena* possesses the core kinase module of the human Hippo signaling, which is coincident with the recent data obtained by Chen et al., 2020 [98]. The fact that the *Tetrahymena* genome seems to contain a gene for a Mob4 homolog, and the existence of the interplay between CdaI and ELO1, led Jiang et al. (2019) [32] to propose the existence in *Tetrahymena* of different Hippo signaling routes using different kinases belonging to MST1/2 and NDR/LATS kinase families, or even different Mob molecules. The studies in *Tetrahymena* clearly showed that Mob1 is a critical factor in cell polarity establishment apart from its role in cytokinesis. On the other hand, experiments carried out in the *Stentor coeruleus*, a ciliate that can regenerate an entire cell-organism from a fragment of one cell clearly showed that Mob1 is a critical factor for morphogenesis. In *Stentor* Mob1 was surgically removed from the anterior and posterior pole of the ciliate’s cells in a Mob1 knockdown background. These cells, contrary to controls, were unable to regenerate a normal morphology showing that Mob1 plays a role in the establishment of both anterior and posterior polarity in *Stentor* [144]. Altogether, the studies in ciliates showed that Mob1 is essential for the perpetuation of the complex patterning and axiation of the ciliate’s cell and morphogenesis, that are linked to accurate cell division ultimately required to maintain cell ploidy and genomic stability [7,144].

#### 3.2.2. MOB in Apicomplexa

The alveolate of Apicomplexa phylum includes parasitic obligate intracellular eukaryotes. These unicellular organisms, which are also permanently polarized cells, possess in the anterior pole a distinctive apical complex used for host cell invasion, composed of conoid, polar rings, subpellicular microtubules, and accessory secretory organelles. Apicomplexans also possess a characteristic non-photosynthetic vestigial plastid termed apicoplast, with few exceptions [145,146,147]. Within this phylum, *Toxoplasma gondii* and *Plasmodium* spp. have been most widely studied due to their burden as parasites of veterinary and medical importance, but also as important biological models for other apicomplexans and unique cellular mechanisms, including cell invasion and division [148]. In fact, Apicomplexa cell division is a unique process since they replicate by forming daughter parasites within a mother cell. For example, *T. gondii* divides by internal daughter budding (endodyogeny) forming two daughter cells within the mother parasite. These daughter cells are delimited by an inner membrane complex and associated subpellicular microtubules, and each contains a complete set of apical organelles (conoid, rhoptries, and micronemes), nucleus, mitochondrion, Golgi apparatus, and apicoplast [149,150]. In these parasites, the division plane is coincident with the antero-posterior axis of the old mother cell, whereas in the ciliates is perpendicular. Once daughter cells are mature the maternal apical complex is disassembled, and the daughter parasites emerge from the maternal plasma membrane [150].

*Mob* genes are present and conserved in the genome of apicomplexan parasites like the cyst-forming coccidians *T. gondii*, *Hammondia hammondi*, *Neospora caninum,* and *Besnoitia besnoiti*, in the monoxenic coccidians *Eimeria tenella* and *Eimeria maxima*, and the most basal apicomplexans *Cryptosporidium*, e.g., *C. muris* and *C. parvum*. Current analyses were not able to identify *Mob* genes in the genomes of hematozoans (data from EuPathDB; https://veupathdb.org/veupathdb/app) namely, *Plasmodium* spp., *Babesia* spp., or *Theileria* spp.

Interestingly, the polarized localization in basal bodies (accepted as the ancestors of centrioles) of Mob1 in *Tetrahymena* non-dividing cells has no parallel in *T. gondii*. Indeed, recombinant *T. gondii* MOB1 protein (recTgMOB1) shows a punctate cytoplasmic localization that is excluded from the apical pole (Delgado et al., unpublished), and no staining is found in the *Toxoplasma* centrosome. The overexpression of the recTgMOB1 causes a marked delay in the tachyzoite proliferation rate, the parasitic form of the asexual cycle of the *T. gondii* life cycle responsible for the acute stage of the disease. Also, *T. gondii* large-scale gene expression data show higher levels of MOB1 mRNAs during the sexual development of the parasite compared to the asexual development (microarray and RNAseq data from Fritz et al., 2012 [151], RNAseq data from Ramakrishnan et al., 2019 [152], available at ToxoDB.org). A similar MOB1 expression pattern is detected in *E. tenella* (RNAseq data from Walker et al., 2015 [153] and Reid et al., 2014 [154], available at ToxoDB.org). Concerning the core kinase module of the human Hippo signaling, the HsMST1/2, HsLATS1/2 and HsNDR1/2 kinases that interact with HsMOB1, the analyses of predicted apicomplexan kinomes detected a high number of divergent kinases, but also highly conserved eukaryotic kinase families [155,156]. These analyses identified several AGC kinases, including two NDR kinases, and only one STE kinase in *T. gondii*. The *T. gondii* AGC/NDR kinases are probable homologs to Warts/LATS1/2 and Tricornred/NDR1/2, respectively. Similarly to TgMOB1, these genes present a mRNA expression that steadily increases during its sexual cycle in the cat’s intestinal epithelium and that is significantly higher compared to the respective mRNA levels in *T. gondii* asexual stages (RNAseq data from Ramakrishnan et al., 2019 [152], available at ToxoDB.org). While the single predicted *T. gondii* STE family kinase was not successfully classified into STE subfamilies, it presents the highest homology with the *N. caninum* STE20 kinase, a subfamily that includes Hippo/MST kinases. However, the TgSTE family kinase and NcSTE20 kinase do not possess the Hippo/MST SARAH domain. Additionally, the *T. gondii* kinome appears to lack kinases containing a CNH domain, a domain present in the STE20 kinases Misshapen/MAP4K4/6/7 and Happyhour/MAP4K1/2/3/5. No homologs to Salvador/SAV1, Kibra/KIBRA, Scalloped/TEAD, NF2/Merlin, or YAP/TAZ have been identified in *T. gondii*. However, one of the *T. gondii* genes (TGME49_306220) encodes two WW domains, as is the case of YAP, the most conserved Hippo effector protein. The *T. gondii* genome also encodes four 14-3-3 proteins, all regulators of YAP/TAZ activity. Finally, PKA, a Hippo pathway activator, controls host cell egress in *T. gondii* tachyzoites [157,158].

### 3.3. Non-Alveolate Protozoa

*Mob* genes were identified in other protozoan parasites such as the kinetoplastid *Trypanosoma brucei*, a member of the Euglenozoa phylum, which expresses two closely related MOB1 proteins, TbMOB1A and TbMOB1B (92.4% identity). Hammarton et al. (2005) [159] showed that TbMOB1 exhibits a punctate cytoplasmic localization in the bloodstream form and is required for cytokinesis but not for mitosis in the bloodstream and procyclic forms. This research also demonstrated that TbMOB1A interacts with the *T. brucei* NDR kinase TbPK50, a protein with high homology to the *T. gondii* NDR kinases. More recently, MOB proteins were also detected and are conserved in other kinetoplastids, i.e., *Trypanosoma rangeli*, *Trypanosoma cruzi*, and *Leishmania major* [160,161] and in the fornicatan *Giardia* [162,163,164]. Conserved *Mob1* genes have also been identified in *Trichomonas vaginalis* and *Entamoeba histolytica*, protozoans belonging to the phyla Parabasalia and Amoebozoa, respectively [165,166]. An analysis of the genome of the protozoan and obligate intracellular plant parasite *Plasmodiophora brassicae* also identified a conserved *Mob1* gene [167]. These data clearly indicate that MOB encoding genes and other members of the Hippo signaling pathway are generally present in protozoa parasites.

### 3.4. MOBs: From Unicellular to Multicellular Organisms

We performed a sequence analysis of MOB-like proteins in a group of multicellular and unicellular organisms. The selection of organisms for this analysis was performed to represent the diversity of the eukaryotic tree of life (see legend of Figure 2). Sequences of orthologs of human MOB proteins were obtained for these organisms from the eggNOG 5.0 database [168]. From these sequences we excluded the ones that presented <50% identity in the amino acids conserved in MOB proteins [169] after Muscle alignment [170]. We ended up with a total of 81 sequences (32 from unicellular organisms and 49 from multicellular organisms) that were analyzed in terms of amino acid identity (see Table 2, Table 3, Table 4, Table 5, Table 6 and Table 7).

**Figure 4:**
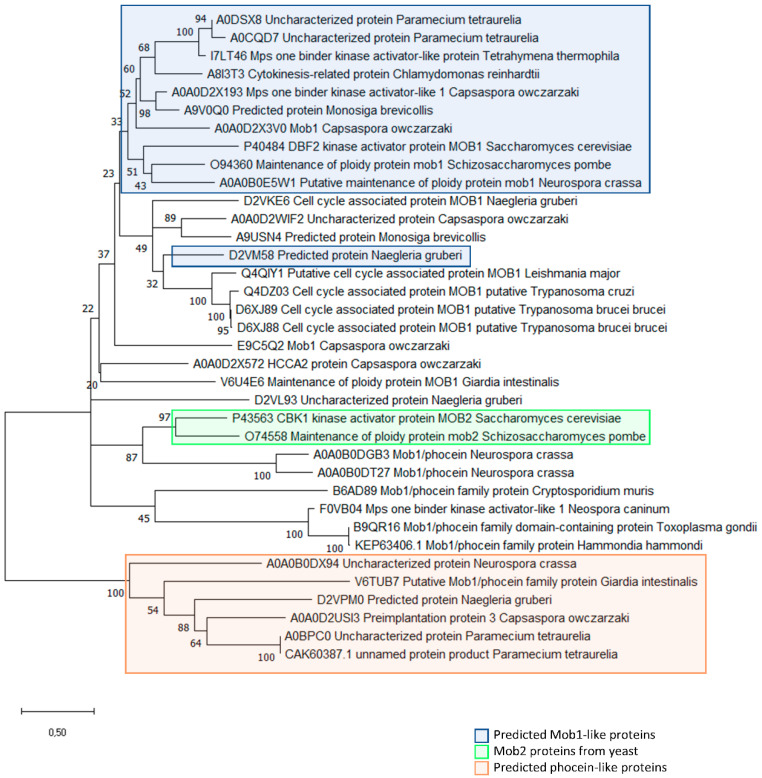
Evolutionary analysis of MOB proteins in unicellular organisms and the multicellular fungi *Neurospora crassa.* The evolutionary history of MOB proteins was inferred by using the Maximum Likelihood method and Le_Gascuel_2008 model [171]. The tree with the highest log likelihood (-9063.20) is shown. The percentage of trees in which the associated taxa clustered together is shown next to the branches. Initial tree(s) for the heuristic search were obtained automatically by applying Neighbor-Join and BioNJ algorithms to a matrix of pairwise distances estimated using the JTT model, and then selecting the topology with superior log likelihood value. A discrete Gamma distribution was used to model evolutionary rate differences among sites (5 categories (+*G*, parameter = 3.0458)). The rate variation model allowed for some sites to be evolutionarily invariable ([+*I*], 0.60% sites). The tree is drawn to scale, with branch lengths measured in the number of substitutions per site. This analysis involved 36 amino acid sequences. All positions with less than 95% site coverage were eliminated, i.e., fewer than 5% alignment gaps, missing data, and ambiguous bases were allowed at any position (partial deletion option). A total of 168 positions was included in the final dataset. Evolutionary analyses were conducted in MEGA X [172]. The sequences included predicted MOB proteins from the chosen unicellular organisms plus the sequences from the multicellular fungus *Neurospora crassa*. Blue rectangle highlights predicted MOB1-like proteins based on >50% amino acid identity among the amino acids conserved in MOB1 proteins [169]. Green rectangle highlights yeast Mob2 proteins. Orange rectangle highlights predicted Phocein-like proteins based on <50% amino acid identity among the amino acids conserved in non-Phocein MOB proteins [169]. The MOB protein sequences for the analysis were obtained for the following organisms: *Amphimedon queenslandica; Arabidopsis thaliana; Branchiostoma floridae; Caenorhabditis elegans; Capsaspora owczarzaki; Chlamydomonas reinhardtii; Ciona intestinalis; Cryptosporidium parvum; Danio rerio; Drosophila melanogaster; Emericella nidulans; Giardia intestinalis; Hammondia hammondi; Homo sapiens; Leishmania major; Monosiga brevicollis; Naegleria gruberi; Nematostella vectensis; Neospora caninum; Neurospora crassa; Paramecium tetraurelia; Saccharomyces cerevisiae; Schizosaccharomyces pombe; Strongylocentrotus purpuratus; Tetrahymena thermophila; Toxoplasma gondii; Trichoplax adhaerens; Trypanosoma brucei; Trypanosoma cruzi.*

The analysis of Table 2 shows that unicellular organisms present a similar degree of identity in the amino acids that are conserved in all MOB family proteins, as described by Stavridi (2003) [169]. Interestingly, the degree of conservation between multicellular and unicellular MOB proteins is also present when we analyze MOB1-like and Phocein-like proteins and their specific conserved amino acid residues with very few exceptions (see Table 3 and Table 4). Phocein proteins are the most divergent and least studied class of MOB proteins. Phocein shares the phocein domain with the rest of the MOB proteins and is a component of the STRIPAK complex [5]. The obtained results show that, when different MOB protein families are analyzed, their degree of conservation in specific residues is maintained throughout evolution, strongly support the possibility that core MOB functions have been maintained. The main difference found between these two groups of organisms is the simultaneous presence of MOB1-like and Phocein-like proteins. In multicellular organisms, both MOB1-like and Phocein-like proteins seem to be present in most of the organisms (the exceptions are *A. thaliana*, *C. intestinalis*, and *N. vectensis* that seem to lack Phocein-like proteins, and *B. floridae* and *C. intestinalis* that seem to lack MOB1-like proteins). Some authors have even suggested that Phocein has conserved functions across multicellular organisms [5]. In our analysis we show that although in unicellular organisms Phocein-like proteins are more uncommon (they are only present in *C. owczarzaki*, *G. intestinalis*, and *Paramecium tetraurelia*), they exist. Also, MOB1-like proteins do not seem to be present in all organisms (e.g., *C. parvum*, *G. intestinalis*, *H. hammondi*, *L. major*, *N. caninum*, *T. gondii*, *T. brucei*, *T. cruzi*) (see Figure 4). Several of these unicellular organisms seem to lack both MOB1-like and Phocein-like proteins (*C. parvum*, *H. hammondi*, *L. major*, *N. caninum*, *T. gondii*, *T. brucei*, and *T. cruzi*) while in the multicellular organisms this only happens in *C. intestinalis*. This shows that increased complexity in the cellular organization seems to be accompanied by the diversification of MOB proteins present in the same organism. It is also interesting to notice that the unicellular organisms that present neither MOB1-like nor Phocein-like MOB proteins can be divided into two groups. The first group composed of *T. cruzi*, *T. brucei*, and *L. major* presents MOB proteins that are more related to MOB1 proteins, while the other group of organisms, composed of *T. gondii*, *H. hammondi*, *N. caninum*, and *C. parvum*, presents MOB proteins more closely related to the MOB2 proteins from yeasts (Figure 4). Also of note is the fact that, although *N. crassa* is a multicellular fungus, its MOB proteins present a high degree of similarity with the proteins of *S. cerevisiae* and *S. pombe* (Figure 4). This led us to postulate that, in fungi, the transition into multicellularity does not seem to be a significant hallmark in MOB proteins diversification. This is also evident when we analyze the unicellular and multicellular sequences together: fungi sequences group together and apart from the other organisms, with some sequences from unicellular organisms (e.g., *Capsaspora owczarzaki)* grouping closer to the ones from multicellular than the fungi do (data not shown).

We can assume that the functional conservation of MOB proteins can also be assessed by the degree of conservation of amino acid residues in structural and regulatory domains [169]. This conservation is particularly evident when we analyze the four amino acid residues that are described to be involved in chelating zinc atom that stabilizes the MOB structure [169]. These four residues are conserved in all sequences that were analyzed, except for the first two cysteines C79 and C84 (the numbering corresponds to the amino acid sequence of HsMOB1A) that are not conserved in *S. cerevisiae* Mob2p and C84 that is also not conserved in one sequence of *N. gruberi* (Table 5). Regarding, the amino acid residues that have been described as targets for regulatory post-translational modifications [75], we also observed a high degree of similarity comparing unicellular or multicellular organisms MOB proteins, especially if we consider MOB1-like proteins. The amino acid residues targeted for phosphorylation on HsMOB1 present similar degree of conservation in MOB1-like proteins from both unicellular and multicellular organisms. An exception is the residue of T12 that is not present in the unicellular organisms. The degree of conservation is particularly relevant for T35 and S38 residues (Table 6). The phosphorylation sites of HsMOB4/Phocein are much more conserved in multicellular organisms in comparison to unicellular organisms’ Phocein-like proteins (Table 7). The fact that these regulatory residues seem to be more conserved in multicellular organisms, associated to the fact that Phocein-like proteins are less represented in unicellular organisms suggests that Phocein-like proteins have evolved and later gained relevant functions in the multicellular organisms. The only unicellular organism that presents conserved phosphorylation sites in its Phocein-like proteins is the ciliate *P. tetraurelia*.

## 4. Concluding Remarks

### 4.1. The Ancient MOB: In the Crossroad of Accurate Cell Division, Cytokinesis, and Morphogenesis

Functional conservation of MOB proteins through the eukaryotic lineage strongly indicates an early emergence in evolution. As evolution progressed, the ancestral functions of these proteins were integrated into more complex signaling pathways to maximize their capacity to cope with the increasing complexity of eukaryotic cells in a multicellular environment.

**Figure 5:**
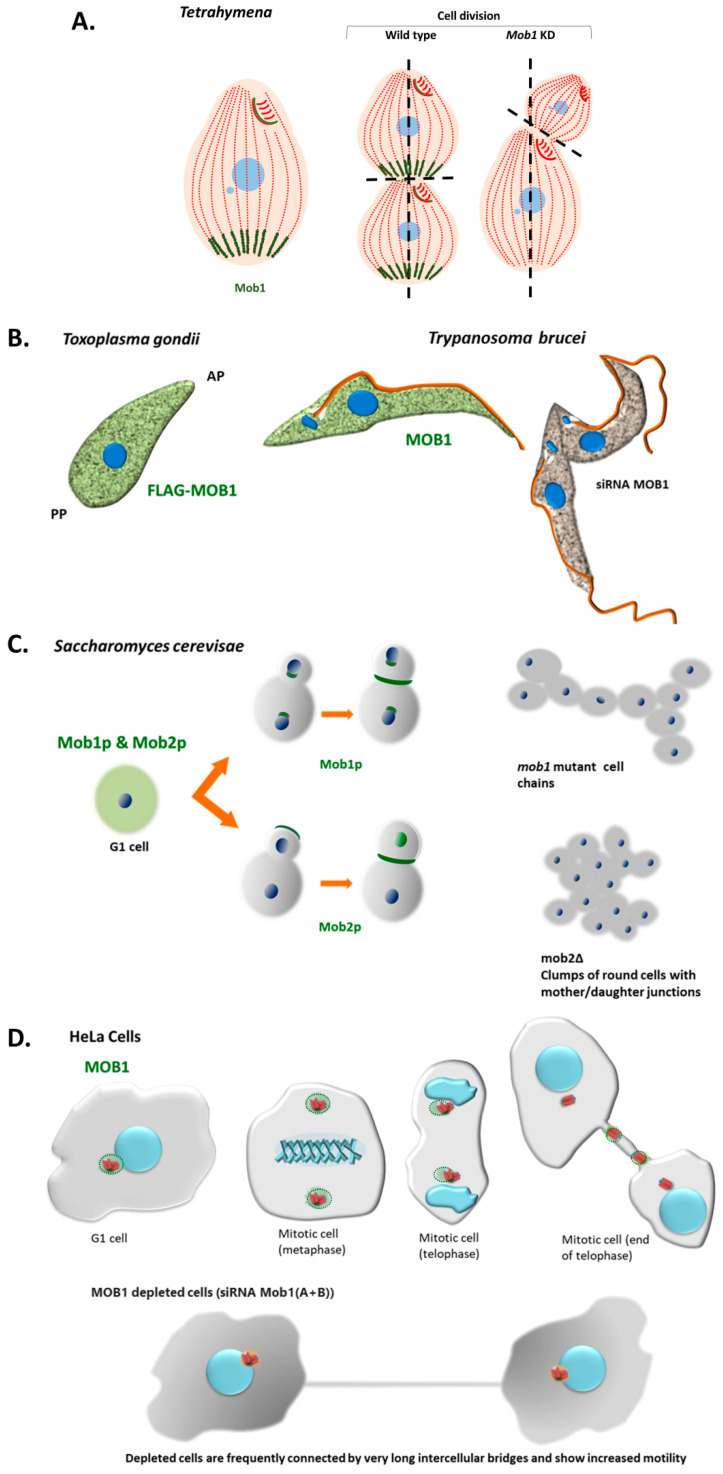
Localization of MOB proteins in different unicellular organisms in comparison to human cells. (**A**) In the ciliate *Tetrahymena* Mob1 accumulates in basal bodies at the posterior pole of the cell forming a gradient through the anterior–posterior axis. During cell division Mob1 is recruited to the basal bodies at the equatorial zone where the cleavage furrow will be established [7]. Mob1 depletion causes the abnormal establishment of the cell division plane and cytokinesis is arrested [7]. (**B**) In *T. gondii*, TgMOB1 protein shows a punctate cytoplasmic localization that is excluded from the apical pole (Delgado et al., unpublished). TbMOB1 in the bloodstream form of *T. brucei* presents a punctate distribution in the cytoplasm throughout the cell cycle. TbMOB1 was shown to be essential in both bloodstream and procyclic life cycle stages of the protozoan. Down-regulation of TbMOB1 in the bloodstream form results in a delay in cytokinesis and leads to a de-regulation of the cell cycle (not shown in the scheme) [159]. In the procyclic form, it affects the accuracy of cytokinesis, presenting mispositioning of the cleavage furrow and incorrect cytokinesis [159]. AP—apical pole; PP—posterior pole. (**C**) In budding yeast Mob1p localizes diffusely to the cytoplasm in G1, S, and G2 phases. In mitotic cells Mob1p first localizes to the spindle pole bodies during mid-anaphase and to a ring at the bud neck just before and during cytokinesis. Mob1p is required for cytokinesis in addition to mitotic exit [108]. Cultures of mutant mob1 strains present cellular chains indicating the role of Mob1p in cytokinesis [108]. In mitotic cells Mob2p first localizes to the growing bud tip and to the bud neck and the daughter nucleus in late mitosis. Mob2p is required for maintenance of polarized cell growth and for mother/daughter separation after cytokinesis. mob2Δ strains grow as clumps of round cells joined at certain regions [131]. (**D**) In human HeLa cells HsMOB1 localizes to the centrosome and this staining pattern remains until the centrioles duplicate and centrosomes separate [55]. In late telophase cells with two separated centrioles, HsMOB1 is detected just in the stronger GFP-centrin signal (probably mother centriole), often the one closer to the midbody. Depletion of HsMOB1A and HsMOB1B by RNAi causes abscission failure and increases cell motility after cytokinesis inducing persistent centriole separation in G1 phase [55].

MOB proteins are required for accurate cell division, ensuring correct genome segregation and distribution of cell structures and compartments between daughter cells. This is accomplished by placing a specific quantity of MOB protein exactly at the cellular furrow region, as indicated by studies in yeast and ciliates [7,32,142,144], but also in human cells [13,48,55,56]. Indeed, the absence of correct MOB protein levels at the furrow region compromises the positioning of the division plane and the mechanism of abscission, i.e., cytokinesis, being this protein a cell hallmark of the division site (Figure 5).

Several studies showed that the perpetuation of complex cell patterning requires the presence of MOB proteins, indicating that its role in cell division is linked to the maintenance of cell architecture, spatial organization (polarity), and, therefore, morphogenesis. In *S. cerevisiae*, MEN is not activated every time the mitotic spindle is mispositioned, which is under the surveillance of the spindle position checkpoint (SPOC) pathway [173]. The SPOC pathway guarantees that MEN is inactive until the correct orientation of the spindle is accomplished. Also, the molecules involved in establishing cell polarity and microtubules work together to establish the MEN asymmetry [127] and polarity factors such as Rho-like GTPase Cdc42p [174] are required for MEN activation. Consequently, mitosis completion and cytokinesis are dependent on correct spindle orientation that results in correct cell polarity. Interestingly, the yeast spindle pole bodies might be a part of the SPOC sensory mechanism [175].

In most organisms, MOB proteins localize at the centrosome or equivalent structure as the spindle pole bodies in yeasts and basal bodies (the accepted ancestors of centrioles) in the ciliates. Besides MOB proteins, other components of the Hippo signaling pathway associate with these structures. In the last years, centrosomes have been emerging as active structures participating in signaling cascades: signaling molecules involved in different pathways are recruited to centrosomes which concentrate and facilitate their interactions. Additionally, their roles in the organization of the cytoskeleton may also facilitate sensing and translating chemical/mechanical intrinsic signals and/or spatial extrinsic signals. Moreover, the structural and functional asymmetry of centrosomes (mother versus daughter centrioles) is explored in asymmetric cell divisions leading to daughter cells that follow different fates, as in the case of the asymmetric cell division of *Drosophila* male stem cells and neuroblasts [176,177]. In these examples the controlled orientation of the mitotic spindle, that is linked to the intrinsic asymmetry of the centrioles, plays a critical role in the determination of which daughter cell will be kept as a stem cell and which one will differentiate [176,177,178,179]. In human cells, MOB1 apparently concentrates on the mother centriole during late telophase which seems important for abscission and cytokinesis (Figure 5) [55]. Similarly, in yeasts, the polarized localization of the MEN/SIN component, including Mobp, in duplicated spindle pole bodies, seems to be important for SIN/MEN regulation and to shut off the signal cascade after cytokinesis completion [124,125]. In *Tetrahymena*, Mob accumulates at the posterior cilia basal bodies defining a specific territory of these structures that is required for cytokinesis and the perpetuation of cell patterning [7]. Therefore, it is tempting to speculate that the polarized localization of Mob and other components of the Hippo pathway at the centrosomes/basal bodies allows to fine-tune the integration of other signals coming from cell polarity, being this permanently present (as in *Tetrahymena*) or transiently established during division (as in the budding yeast), thus promoting the correct positioning of cell division plane and the perpetuation of cell morphology. These observations raise the possibility that mechanisms required for asymmetric cell division, e.g., in metazoans’ stem cells, may have their origins in systems that regulate cell division in unicellular ancestors.

It is interesting to verify that in the protozoan parasites *T. gondii* and *T. brucei*, both permanently polarized cells, MOB proteins do not accumulate at the centrosome and/or basal bodies (Figure 5). These differences may be related to oddities observed during cell division of these organisms, main structural/functional differences of their centrosomes, the existence of other prominent microtubule organizing centers in these cells, and/or different evolutionary strategies due to its lifestyle. In fact, the *T. gondii* centrosome is composed of a pair of atypical short parallel centrioles (they are 3.5 times shorter than their mammalian counterparts), formed by nine singlets microtubules that are arranged in parallel [180]. *T. gondii* centrosome also seems to lack several important regulatory centrosomal proteins, e.g., PLK4 and PLK1 homologs (for review, see Morlon-Guyot et al., 2017 [180]). In *T. brucei*, although TbMOB1 seems to be required for cytokinesis, the non-specific localization of the protein at the basal bodies is also accompanied by no detection of the protein at the cytokinesis furrow, suggesting that TbMOB1 may not be recruited to the site of cell cleavage to initiate cytokinesis [159]. Moreover, the *T. brucei* NDR kinase TbPK50 seems to be a MOB-dependent kinase that can functionally complement the *S. pombe Orb6* mutant, which may demonstrate that this protein is able to play polarity regulatory functions, at least in yeast [159]. This lead to the proposal that, during evolution, specific requirements of the cytokinesis process in *T. brucei* have coupled TbMOB1 as a regulator of cytokinesis to the cell polarity-controlling kinase TbPK50 [159]. However, TbPK50 also has a cytoplasmic localization suggesting that trypanosomes may have evolved alternative mechanisms for linking accurate division to morphogenesis. It is interesting to note that in our sequence analysis both *T. gondii* and *T. brucei* MOB proteins do not share the conserved amino acid residues with the MOB1 proteins from either multicellular organisms or yeast (see Section 3).

### 4.2. MOB: New Roles, the Same Function?

In the modern signaling pathways involving MOB proteins, signals coming from junctional cell complexes are integrated to control cell proliferation and tissue homeostasis. Interestingly, ortholog genes encoding some of these cell-cell junctions molecules are already found in unicellular organisms [95,181,182]. For example, it has been suggested that the cadherins present in the choanoflagellate *M. brevicollis* play a role in sensing and responding to extracellular cues [183]. This is supported by the analysis of the functional domains present in these cadherins that suggest a link between them and signal transduction pathways, such as tyrosine kinase and hedgehog signaling in animals [182]. Moreover, the *C. owczarzaki* genome also possesses several ortholog genes that are associated with cell junctional complexes and are required for the establishment of apical-basal polarity in epithelial cells and cell migration, e.g., the aPKC kinase or the KIBRA and Lgl proteins [95]. Consequently, we can envisage that MOB containing pathways were already able to respond to environmental challenges in unicellular organisms.

The analysis of MEN/SIN and Hippo components clearly shows that these pathways share core elements that are highly conserved throughout the evolution, namely the central module composed of MST kinases-MOB1-NDR/LATS kinases. This module, both in unicellular and multicellular organisms, concentrates signals that allow linking of correct cell division/cytokinesis to morphogenesis by integrating the information of cell architecture/polarity. However, vertebrate cells explored this ancient module by turning this core into a central hub that integrates a variety of distinct signals, conditions, and stimuli that are now critical for tissue homeostasis and organ development. These signals may be mechanical, originated from cell-cell contacts and cell shape (depending on cell density), from cell interactions with extracellular matrix (microenvironments), from cell adhesion and polarity, and from different stress conditions (for review, see Ma et al., 2019 [19]). These multiple signals are integrated to control the activity of YAP/TAZ effectors that regulate many genes in each cell type. Ma et al. (2019) [19] suggested that YAP/TAZ transcriptional programs should be precisely regulated in a cell context-specific manner since only a small number of genes are commonly regulated by the Hippo pathway among different cell types. The observations that YAP/TAZ activity is mediated by different chromatin complexes, like NCOA6 histone methyltransferase complex or the SWI/SNF chromatin remodeling complex may contribute to explain why YAP/TAZ targets are cell-type dependent [19]. The integration of distinct signals in the Hippo pathway seems also dependent on the ability of MOB proteins to interact with MST and NDR kinases, originating distinctive complexes that may allow cells to integrate those many different signals in the signaling pathway.

As previously mentioned, different MOB isotypes differentially interact with various Hippo and non-Hippo proteins and can regulate other MOB isotypes. HsMOB1 binds to HsMST1/2, HsNDR1/2, HsLATS1/2, and PRAJA2, acting as a canonical Hippo signal regulator while also being regulated by ubiquitin-proteasome degradation [28]. HsMOB2 binds to HsNDR1/2 (but not HsMST1/2 or HsLATS1/2) and HsRAD50 [54]. Its interaction with HsNDR1/2 inhibits HsNDR1/2-MOB1 interaction and favors canonical Hippo signaling, while binding to HsRAD50 promotes DNA damage response. Conversely, HsMOB3 can inhibit HsMST1 signaling and the HsMST4-MOB4/Phocein complex obstructs HsMST1-MOB1 binding, therefore downregulating canonical MOB1 Hippo signaling [34,36]. Furthermore, the same function can be performed by different MOB isotypes in different species or a tissue-specific manner (Figure 3). Fungi MOB proteins with distinct isotypes, NcMOB1, ChMOB2, and SmMOB4/Phocein, all participate in conidiation, ascosporogenesis, and meiosis [3,4,23]. *M. musculus* MOBs show high redundancy in morphogenesis: MmMOB1, MmMOB2, MmMOB4/Phocein are necessary for neuronal development while MmMOB1 also promotes lung morphogenesis [34,53,59,62,64]. Additionally, the signaling pathways involved in similar MOB functions may not coincide, e.g., MmMOB2 interacts with NDR2 while MmMOB4/Phocein is involved STRIPAK complex signaling [34,64]. Overall, different MOB proteins can regulate each other, directly or indirectly, and its functions span across isotypes, which translates into significant MOB functional overlapping in an organism (Figure 2). Redundancy between members in the Hippo pathway is not limited to MOBs, e.g., YAP/TAZ cytoplasmic retention is very significantly diminished by the combined deletion of the GCK kinases MSTs, MAP4Ks, and TAOKs, which indicates an overlap in the role of these kinases involved in canonical and non-canonical Hippo signaling [184,185]. The redundancy between MOBs and other proteins of the Hippo pathway may contribute to the plasticity of the system to cope with multiple signals.

Remarkably, MOBs can also interact directly with non-kinase proteins. MOB1 binds the cell cycle progression PP6 phosphatase and the Rho guanine exchange factors DOCK6-8 which promote actin cytoskeleton polarization signaling via RAC1 [51,52,63]. It is noticeable that the guanine nucleotide exchanger Dock6 is simultaneously specific for the signaling of protein RAC1 and the highly conserved rho-type GTPase CDC42, an upstream regulator of the MEN and a critical factor in cell polarity. MOB2 can interact with the ATPase/helicase RAD50 promoting DNA damage response [54]. Although it is still uncertain how these complexes affect the Hippo signaling pathway, this shows that MOB regulation and activity is much more complex than previously suspected.

The recognition that MOBs integrate distinct complexes, where may not have kinase partners, indicates that we are far from having a complete picture of their cellular functions and the multiplicity of signaling pathways where they participate. Supporting this idea is the fact that MOB and other members of the Hippo signaling pathways have been associated with cilia biogenesis and function. Indeed, the analysis of Mob1 function in the ciliate *Tetrahymena* revealed that Mob1 gene was upregulated in response to ciliogenesis and *Tetrahymena* Mob1-depleted cells presented a delay in reciliation in comparison to control cells [7]. Other MOB interactors like the NDR2 kinase were also implicated in cilium formation by being identified as the causal gene for a canine ciliopathy characterized by early retinal degeneration [186,187]. Later it was demonstrated that the NDR2 kinase plays a critical role in ciliogenesis through Rabin-8 phosphorylation by causing a switch in binding specificity of Rabin8 from phosphatidylserine to Sec15, which promotes local activation of Rab8 and ciliary membrane formation [50]. NDR kinases and Hippo signaling members, like MOB1, seem to have a preponderant role in the differentiation/maintenance of retinal photoreceptors where the connector cilium links the body of the cell to the outer segment. In fact, altered expressions of MOB1 have been observed in early retinal degeneration [41]. Other MOB interacting kinases like MST1/2 (Hippo) kinases localize to the basal body and are required for ciliogenesis [188]. Furthermore, MST1/2-SAV1 associates with the NPHP complex (proteins encoded by genes that are a hot spot for mutations in the nephronophthisis (NPHP)-related ciliopathic disorders), that regulates the loading of cargoes into intraflagellar transport (IFT) complexes at the cilium transition zone, and promotes cilia assembly [189]. These proteins have been also pointed as regulators of the Hippo pathway [190,191,192]. Another example of the link between MOB/Hippo signaling and cilia is the EXOC5 (exocyst trafficking complex) involved in ciliogenesis during retinal development. EXOC5 knockout causes increased phosphorylation of MOB, Hippo pathway activation, and loss of cilia accompanied by photoreceptor outer segment degeneration, most likely due to affected traffic in connector cilia of outer-segment proteins [193]. The knockdown of MOB2 within the developing mouse cortex affects cilia positioning and number within migrating neurons [59].

From these data and the results in ciliates, MOB1 seems to be one of the important components of the Hippo pathway that bridges this signaling pathway to ciliogenesis. In ciliates there are cell territories where basal bodies are enriched in MOB1 and other members of the Hippo signaling pathway. Therefore, we speculate that, in these basal body populations, MOB1 allows the concentration of intrinsic and/or extrinsic signals received by motile cilia, which are also signaling organelles. The signals concentrated in these specific basal bodies will be locally transduced creating signal asymmetries inside a single cell that parallels development in multicellular organisms.

The ancestry of MOB proteins clearly indicates their importance from unicellular to multicellular organisms, coupling the mechanisms underlying accurate cell division, cell number, polarity, and morphogenesis (which became fundamental in metazoan development) to tissues/organs homeostasis. The recent reports that MOBs are associated with cilia, critical organelles in signaling transduction, show that early in evolution cell division and maintenance of cell architecture were in crosstalk with environmental conditions. The evolution of these signaling pathways may have favored distinct cells in a population to harmonize their functions and numbers in response to environmental conditions. This probably contributed to launch the foundations for multicellularity. In multicellular organisms, the role of core components of these pathways was expanded to cope with increased complexity, new multiple signals, and environments. The study of MOB proteins from unicellular organisms clarified the intrinsic role of MOB in cell polarity and cell pattern perpetuation. The more recent discoveries concerning MOB as a member of new distinct complexes will probably challenge our understanding of how these proteins became crucial for complex multicellular organisms’ homeostasis and how their malfunction leads to disease.

## Figures and Tables

**Table 1:** MOB functions in multicellular organisms.

Protein		Described Functions	Functional Category				References
			TH	M	D	CC	
**Mammals and birds**	HsMOB1	Tissue growth (suppressor); apoptotic signaling; mitotic exit; protein complex positioning to spindle midzone, mitotic spindle orientation; centrosome duplication and disjunction; cytokinesis	X			X	[13,14,27,28,29,30,34,48,55,56,57]
	HsMOB2	Tissue growth (suppressor); cortical development; mitotic spindle orientation; centrosome duplication; DNA damage response	X	X		X	[14,33,48,54,58,59,60,61]
	HsMOB3	Tissue growth (enhancer through MST1 inhibition); apoptotic signaling	X			X	[14,36]
	HsMOB4	Tissue growth (enhancer through MST1-MOB1 complex inhibition)	X				[34,35]
	MmMOB1	Tissue growth (suppressor); lung and neuronal morphogenesis; stem cell differentiation and maintenance; centrosome duplication; actin cytoskeleton polarization	X	X	X	X	[10,37,38,39,40,53,62,63]
	MmMOB2	Neuronal and cortical development; actin cytoskeleton organization.		X			[59,64]
	MmMOB4	Dendritic arborization in neuronal development		X			[34]
	CfMOB1	Photoreceptor growth; mitosis	X			X	[41]
	GgMOB2	Tissue growth (suppressor); follicle development	X	X			[42]
**Insects**	DmMOB1	Tissue growth (suppressor); stem cell differentiation; chromosome segregation	X		X	X	[2,15,26,62,65]
	DmMOB2	Wing hair, photoreceptor and neuromuscular junction morphogenesis		X			[66,67,68]
	DmMOB4	Synapse morphogenesis and microtubule organization; spindle pole assembly; axonal transport of autophagosomes		X		X	[69,70,71]
**Plants**	AtMOB1	Plant growth (enhancer); development of stem and root; sporogenesis and gametogenesis; stem cell differentiation and maintenance; apoptotic signaling; cytokinesis	X	X	X	X	[43,44,45,46]
	MsMOB1	Tissue growth (suppressor); stem cell association	X		X		[47,72]
**Fungi**	NcMOB1	Tissue growth (enhancer); septum, aerial mycelium and fruiting body development; conidiation; ascosporogenesis; meiosis	X	X	X	X	[3]
	NcMOB2	Tissue growth (enhancer); hypha development; conidiation	X	X	X		[3]
	NcMOB4	Tissue growth (enhancer); fruiting body development; vegetative cell fusion	X	X			[3,24]
	AnMOB4	Tissue growth (suppressor); ascosporogenesis; meiosis.	X		X	X	[22]
	SmMOB4	Vegetative cell fusion; conidiation and ascosporogenesis; meiosis		X	X	X	[23]
	ChMOB2	Conidiation and ascosporogenesis; meiosis			X	X	[4]

TH—tissue homeostasis, M—morphogenesis, D—differentiation, CC—cell cycle progression, Dm—*Drosophila melanogaster*, Hs—*Homo sapiens*, Mm—*Mus musculus*, Cf—*Canis familiaris*, Gg—*Gallus gallus*, At—*Arabidopsis thaliana*, Ms—*Medicago sativa*, Nc—*Neurospora crassa*, An—*Aspergillus nidulans*, Sm—*Sordaria macrospora*, Ch—*Colletotrichum higginsianum*.

**Table 2:** Identity in amino acid residues that are conserved in all MOB family proteins.

	P48	D52	W56	N69	M87	A89	A111	Y114	F132	P133	Y163	F186	F189
Identity in MOB proteins (multicellular)	100%	84%	100%	93%	100%	67%	98%	96%	98%	100%	91%	84%	84%
Identity in MOB proteins (unicellular)	97%	67%	94%	64%	92%	78%	100%	92%	92%	89%	92%	89%	86%

The residue numbering corresponds to HsMOB1A protein.

**Table 3:** Identity in amino acid residues that are conserved in MOB1-like proteins.

	E33	T35	G37	S38	A44	E51	D63	Q67	M70	T76	Y92	E93	S110	D126	S182
Identity in MOB1-Like proteins (multicellular)	50%	94%	88%	100%	100%	94%	100%	94%	69%	81%	81%	94%	94%	94%	75%
Identity in MOB1-Like proteins (unicellular)	36%	100%	100%	73%	91%	100%	73%	55%	27%	45%	91%	100%	82%	64%	45%

The residue numbering corresponds to HsMOB1A protein.

**Table 4:** Identity in amino acid residues that are conserved in non phocein-like proteins.

	E55	A59	Y72	W97	D128	F140	P141	F144	N180	H185	E192	L207
Identity in non phocein-like MOB proteins (multicellular)	74%	97%	85%	94%	74%	100%	94%	100%	94%	91%	82%	74%
Identity in non phocein-like MOB proteins (unicellular)	80%	83%	80%	90%	80%	77%	97%	100%	100%	97%	77%	83%

The residue numbering corresponds to MOB1A human protein.

**Table 5:** Identity in amino acid residues that are responsible for zinc binding.

	C79	C84	H161	H166
Identity in MOB proteins (multicellular)	100%	100%	100%	100%
Identity in MOB proteins (unicellular)	97%	94%	100%	100%

The residue numbering corresponds to HsMOB1A protein.

**Table 6:** Identity in amino acid residues that are phosphorylated in HsMOB1.

	T12	Y26	T35	S38	S146
Identity in Mob1-like proteins (multicellular)	75%	63%	94%	100%	44%
Identity in Mob1-like proteins (unicellular)	-	45%	100%	73%	27%

The residue numbering corresponds to HsMOB1A protein.

**Table 7:** Identity in amino acid residues that are phosphorylated in HsMOB4/Phocein.

	Y141	S147
Identity in phocein-like proteins (multicellular)	73%	73%
Identity in phocein-like proteins (unicellular)	0%	40%

The residue numbering corresponds to HsMOB4/Phocein protein.
